# Zinc-vacancy defects in ZnO nanorods induced visible-light activity of photoelectrochemical glucose sensing: experimental and DFT+U analysis

**DOI:** 10.3389/fchem.2026.1856857

**Published:** 2026-06-16

**Authors:** Trung Tin Tran, Huynh Nhu Nguyen Thi, Trung Nghia Tran, Tran Viet Cuong, Quang Vinh Lam

**Affiliations:** 1 Faculty of Physics and Physics Engineering, University of Science, VNUHCM, Ho Chi Minh City, Vietnam; 2 Laboratory of Laser Technology, Faculty of Applied Science, Ho Chi Minh City University of Technology (HCMUT), Ho Chi Minh City, Vietnam; 3 Vietnam National University Ho Chi Minh City, Ho Chi Minh City, Vietnam; 4 VKTECH Research Center, NTT Hi-Tech Institute, Nguyen Tat Thanh University, Ho Chi Minh City, Vietnam; 5 Nguyen Tat Thanh University Center for Hi-Tech Development, Saigon Hi-Tech Park, Ho Chi Minh City, Vietnam

**Keywords:** DFT+U, electrodeposition, electronic structure, non-enzymatic glucose sensor, photoelectrochemical, ZnO nanorods

## Abstract

Non-enzymatic photoelectrochemical (PEC) glucose sensing using ZnO nanorods (ZnO NRs) is typically constrained to UV excitation, as stoichiometric ZnO possesses a wide band gap. In this work, this UV-only limitation is addressed by deliberately exploiting intrinsic defect chemistry: zinc-vacancy (VZn) defects generated during low-temperature electrodeposition are engineered as the central design knob to activate visible-light PEC glucose sensing—without intentional doping, noble-metal decoration, or multi-component heterostructures. Vertically aligned ZnO NRs grown on fluorine-doped tin oxide (FTO) retain the wurtzite structure but exhibit a reduced optical band gap of ∼2.32 eV (UV–Vis/Tauc), consistent with defect-related electronic states. Under 460 nm illumination in phosphate buffer (pH 7.4), the ZnO NRs/FTO electrode delivers a stable, concentration-dependent photocurrent for glucose oxidation with a linear range of 0.5–10 mM and a detection limit of 12.5 μM. A synergistic combination of spatially resolved EDX compositional analysis and DFT + U electronic-structure cal-culations demonstrates that VZn induces localized band-tail/sub-bandgap states near the band edges, lowering the effective excitation energy and facilitating carrier generation and interfacial charge transfer under visible light. These results establish intrinsic zinc-vacancy engineering as a scalable, metal-free strategy to push ZnO-based non-enzymatic PEC glucose sensing from UV-only operation toward solar-compatible, visible-light activation.

## 1 Introduction

Diabetes mellitus, a chronic metabolic condition characterized by persistently high blood glucose, is a major global health concern (Ogurtsova et al., 2022). Despite advancements in diabetes management, there remains a pressing need for non-invasive, reliable glucose monitoring technologies. To approach this, non-enzymatic electrochemical (EC) glucose sensors show promising development prospects due to their improved stability, lower costs, and simpler fabrication processes. Metal-oxide-based glucose sensors using materials such as ZnO, CuO, and NiO have therefore been extensively investigated; however, their practical performance is still limited by well-known challenges, including ZnO’s chemical instability under fluctuating pH and its predominantly UV-active wide band gap, NiO’s relatively high working potential and often limited linear detection range, and selectivity issues caused by interference from other electroactive species (Ahmad et al., 2017; Baru et al., 2021; Chitare et al., 2021; Dong et al., 2021; Singer et al., 2020). The combination of these materials are also utilized to improve the required characteristics of the sensing system. For instance, Haghparas et al. reported the high sensitivity EC glucose sensor system using modified structure of dumbbell-shaped double-shelled hollow nanoporous CuO/ZnO with the linear range of 500 nM - 100 mM and the sensitivity of 1,536.80 μA mM^−1^ cm^−2^ (Haghparas et al., 2021).

Beside the EC-typed glucose sensors, photoelectrochemical (PEC) technology, which leverages light energy to enhance glucose detection, has gained significant attention owing to its ease of use, sensitivity, and specificity. The PEC process involves the interaction of the analyte with a photosensitive material or electrolyte, resulting in a measurable change in the PEC signal, enabling precise glucose quantification. Due to the ability of light absorption, metal oxide based semiconductive materials are also utilized for glucose sensing applications (Chitare et al., 2021; Siavash et al., 2024; Tobaldi et al., 2020). Among them, ZnO is a promising material for PEC glucose sensing, as demonstrated in this work by a clear glucose-dependent photoelectrochemical response under visible-light illumination. Various ZnO nanostructures, including nanorods (NRs), nanowires, nanoflowers, and nanoparticles, have been synthesized using methods like hydrothermal synthesis, sol-gel, and chemical vapor deposition. These synthesis methods offer control over ZnO nanostructure morphology, size, and surface characteristics, which significantly influence their electrocatalytic activity for PEC glucose detection (Bakranova et al., 2023; Lee et al., 2011; Tao et al., 2023).

In this study, ZnO nanorods were synthesized by low-temperature electrodeposition and characterized using structural and morphological techniques. Their photoelectrochemical response was investigated under light irradiation. Importantly, electrodeposition parameters such as the applied voltage (1–3 V), deposition time (20–40 min), and solution temperature (80 °C) were found to play a critical role in governing both nanorod growth kinetics and intrinsic defect formation. Under relatively low-temperature and kinetically limited growth conditions, non-equilibrium incorporation of intrinsic point defects—particularly zinc vacancies is favored, consistent with first-principles defect thermodynamics identifying V_Zn_ as a dominant intrinsic acceptor in ZnO under O-rich or non-equilibrium growth conditions (Janotti et al., 2009; Janotti and Van de Walle, 2007). Experimental confirmation of such Zn-deficient defects is provided by the spatially resolved EDX analysis along individual nanorods, which reveals a systematic reduction in the Zn/O atomic ratio from the base to the tip. These observations are consistent with previous reports on low-temperature solution/electrochemical growth of ZnO, where growth parameters strongly influence vacancy concentration and defect-mediated electronic properties (Janotti et al., 2009; Janotti and Van de Walle, 2007; Oba et al., 2008).

First-principles calculations based on Density Functional Theory have been widely used to investigate the electronic properties of semiconductor materials. Nevertheless, conventional local/semi-local functionals (e.g., LDA/GGA) often underestimate the bandgap and misrepresent the Zn 3d–O 2p hybridization and localized electronic states in ZnO (Janotti et al., 2009; Lany and Zunger, 2010). To overcome this limitation, the Hubbard U correction is commonly introduced to better account for the on-site Coulomb interaction of Zn 3d electrons (and, in some implementations, O 2p states) within the DFT + U framework (Anisimov et al., 1991; Oba et al., 2008). In this work, DFT + U calculations were carried out using the PBE–GGA functional. The Hubbard correction was applied to Zn 3d and O 2p states; after testing several (U_d_, U_p_) combinations, benchmarking against the experimental band gap of ZnO (∼3.3 eV) indicated that U_d_ = 7 eV and U_p_ = 8 eV yield a ZnO band gap (3.31 eV) closest to the experimental value, consistent with prior DFT + U reports, and these values were then used consistently in all subsequent pristine and V_Zn_-defective calculations (Haruna et al., 2020; Larsen et al., 2017).

## 2 Materials and methods

### 2.1 Chemicals

Chemicals, including Zinc nitrate hexahydrate (Zn(NO_3_)_2_.6H_2_O) and Hexamethylene tetramine (C_6_H_12_N_4_) were utilized for the electrodeposition synthesis of ZnO NRs. D-Glucose (C_6_H_12_O_6_.H_2_O) was dissolved in Phosphate-buffered saline (PBS, pH 7.4) for EC and PEC experiment. All reagents were of ACS grade and obtained from Merck (Germany) with a purity of 99%.

### 2.2 Apparatus

The synthesis of ZnO NRs via the electrodeposition method was utilized by a DC power supply KEYSIGHT E3632A. A platinum wire (diameter 0.5 mm - Redox Me AB, Sweden) was employed as the Counter Electrode for electrodeposition process, while FTO electrode acting as Working Electrode (WE) from Redox Me AB, Sweden.

The crystal structure and surface morphology of the sample were assessed by X-ray diffraction (XRD, D8 Advance Eco, Germany) and scanning electron microscopy (SEM, Hitachi S-4800, Japan), respectively. High-resolution transmission electron microscopy (HRTEM) was employed to examine the crystallinity, lattice fringes, and microstructural features of individual ZnO nanorods, while energy-dispersive X-ray spectroscopy (EDX) was used to determine the elemental composition and local Zn/O atomic ratio at different positions along the nanorods (TEM, JEOL JEM-2100F). The optical characteristics were investigated by ultraviolet–visible absorption spectra (UV–Vis) using Agilent Cary 4000. The pH values of the solutions were adjusted using a Hi98115 Hanna pH meter.

All EC and PEC experiments were conducted on a DY2100 mini potentiostat (Digi-Ivy Instrument Company, USA) with a rhodium-plated counter electrode (CE, Redox Me AB, Sweden) and Ag/AgCl reference electrode (RE, Redox Me AB, Sweden). The excitation light is a 460 nm LED with the intensity of 50 mW/cm^2^.

### 2.3 Preparation of the ZnO NRs/FTO for Working Electrode

**FIGURE 1:**
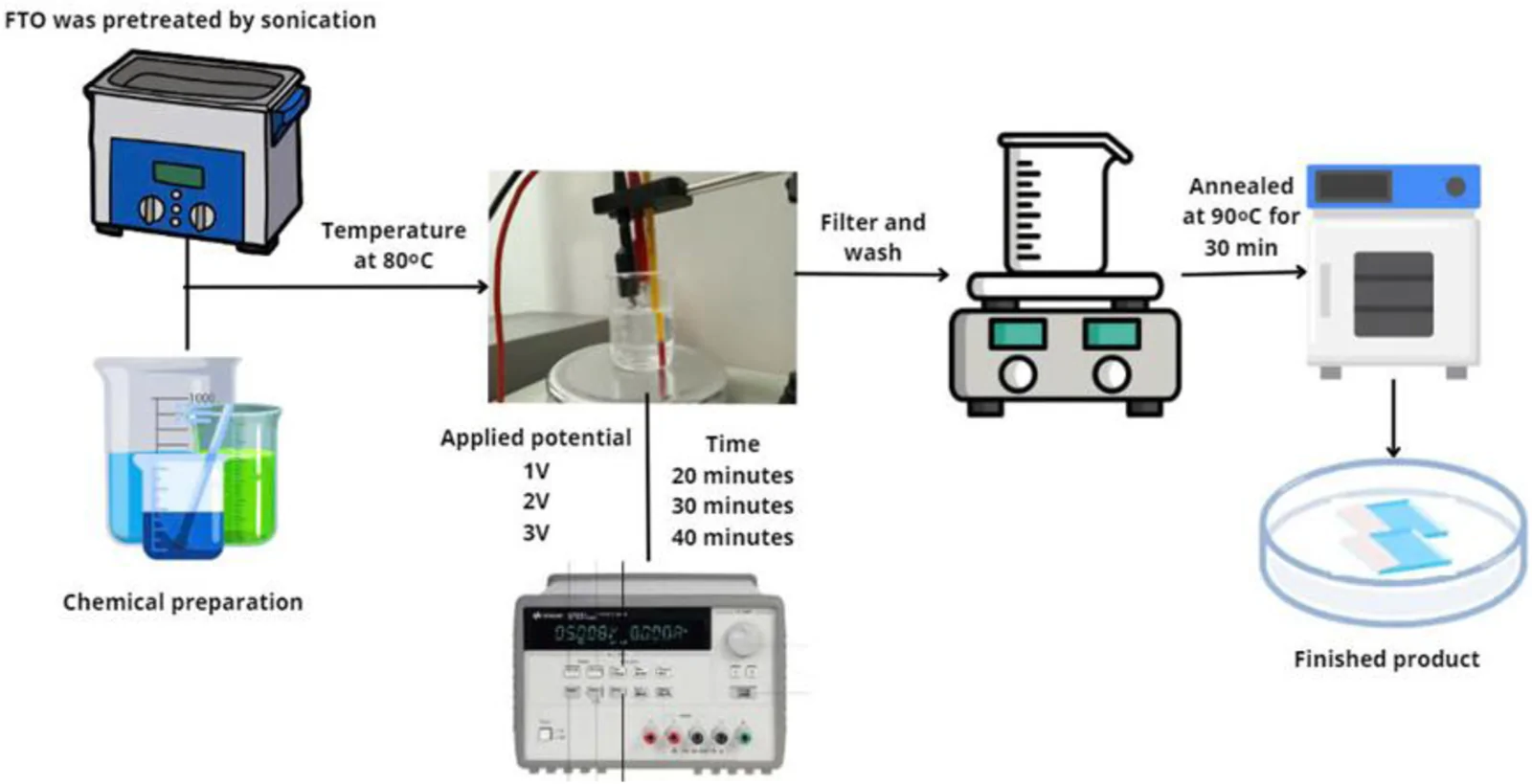
Flowchart of the ZnO NRs synthesis process using electrodeposition method.

**FIGURE 2:**
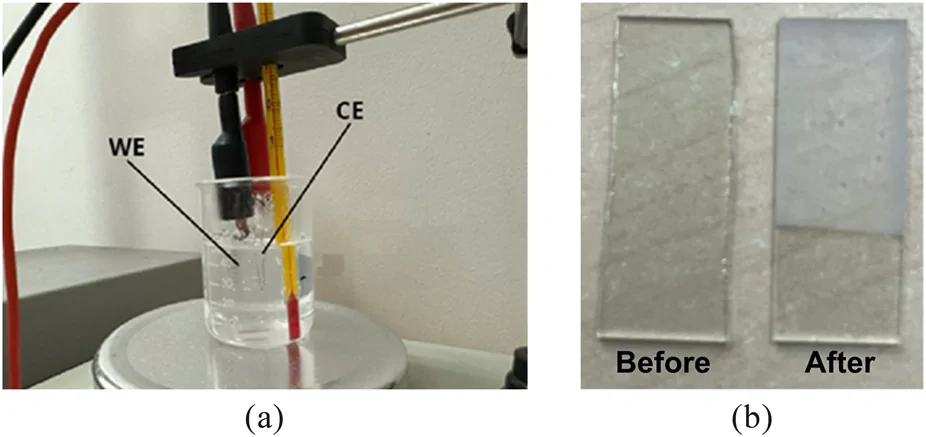
Electrodeposition set-up for simplified 2-electrode system and its result: **(a)** two-electrode config for electrodeposition system, **(b)** the FTO electrodes before and after electrodeposition process.

Figure 1 shows the electrodeposition procedure used for ZnO NRs/FTO. Firstly, dissolve 0.3 g of Zn(NO_3_)_2_.6H_2_O and 0.14 g of C_6_H_12_N_4_ in 400 mL of distilled water. Simultaneously, FTO electrode with the size of 2.5 cm × 0.5 cm was cleaned using ultrasonication for 15 min in a mixture of acetone, ethanol, and distilled water with a ratio of 1:1:1. After that, the FTO was used as WE and connected to the negative terminal while CE was connected to the positive terminal. Two electrodes were placed 3 cm apart in the prepared solution (Figure 2a). The applied voltage was set at three different levels: 1 V, 2 V, and 3 V, and the electrodeposition time was varied between 20, 30, and 40 min to determine the optimal conditions. The temperature was controlled at 80 °C during the deposition process. After the designated time, the sample was removed from the electrolyte solution and rinsed with distilled water in stirring machine for 2 min to remove any excess solution. Finally, the sample was dried at 90 °C for 30 min. The image of FTO electrodes before and after electrodeposition process is shown on Figure 2b.

### 2.4 Photoelectrochemical investigations

**FIGURE 3:**
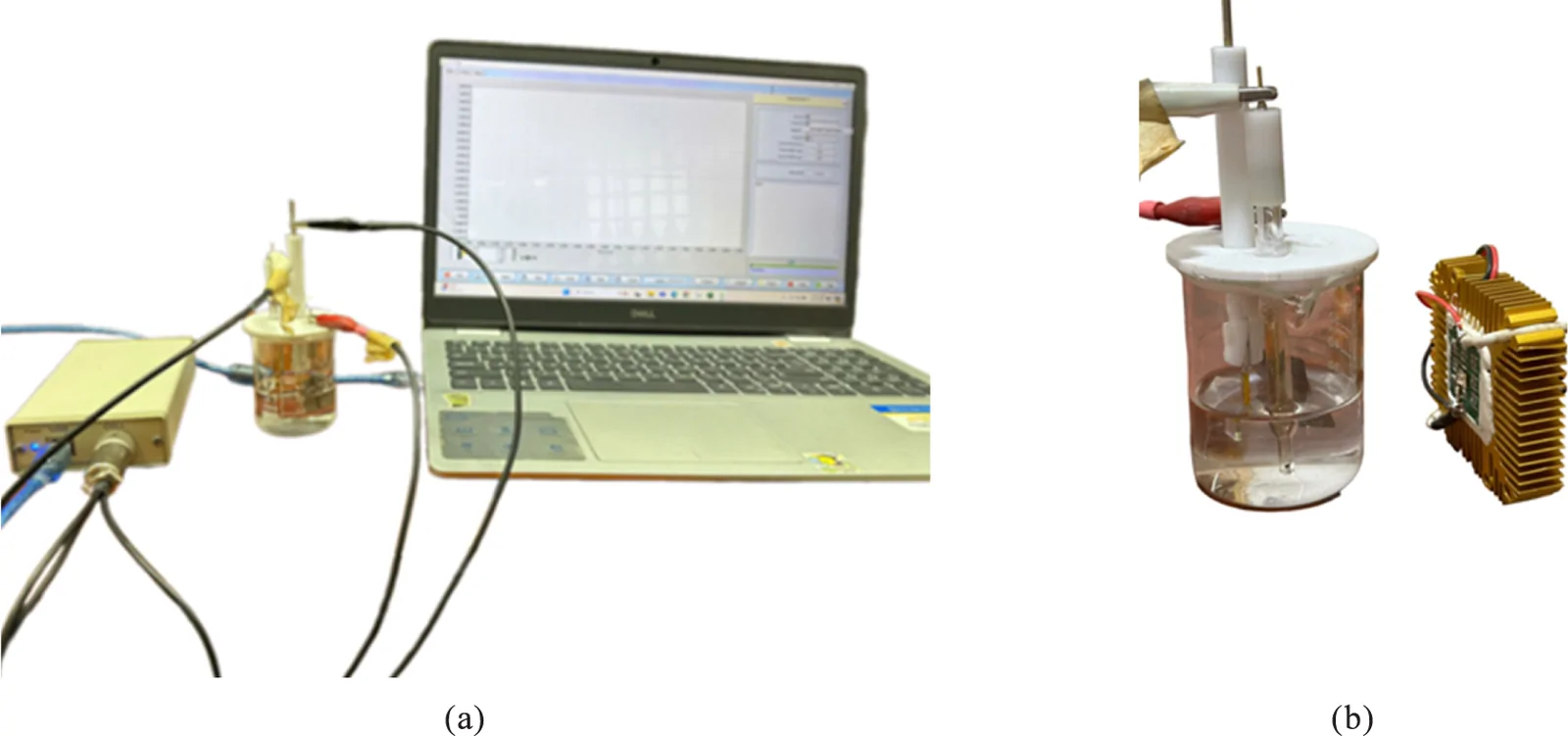
Experimental setup for **(a)** EC and **(b)** PEC measurements.

Figure 3 illustrates the experimental setup of the PEC system, including the electrode cover. Prior to PEC measurements, cyclic voltammetry (CV) was conducted at 0.05 V/s in 3 mM glucose PBS to assess the reactivity of FTO and ZnO NRs/FTO electrodes and determine the redox potential. Subsequently, the PEC sensor was operated in chronoamperometric mode at the oxidation potential identified in CV, with a sensitivity setting of 10^-4^ A/V. The excitation light was cycled on and off every 4 s. The PEC performance of ZnO NRs/FTO electrodes was evaluated with glucose concentrations ranging from 0.5 to 10 mM in PBS. All electrodes were thoroughly rinsed between measurements with subsequent glucose solutions.

### 2.5 Density Functional Theory calculations

To gain deeper insight into the electronic structure of ZnO and to support the experimental observations, first-principles calculations were performed within the framework of Density Functional Theory.

The calculations were performed using the Quantum ESPRESSO package within the framework of Density Functional Theory (DFT). Structure construction and input generation were carried out using the Atomic Simulation Environment (ASE) Python package (Larsen et al., 2017). The exchange–correlation interaction was treated using the generalized gradient approximation with the Perdew–Burke–Ernzerhof functional (PBE–GGA). A plane-wave basis set was employed with a kinetic-energy cutoff of 35 Ry. The Brillouin zone was sampled using a Monkhorst–Pack k-point mesh of 7 × 7 × 2.

Convergence of the plane-wave cutoff energy and Brillouin-zone sampling was verified by total-energy tests (Table 1). As summarized in Table 1, the total energy varies only negligibly upon increasing the cutoff beyond 35 Ry and refining the k-point mesh beyond 7 × 7 × 2; therefore, these settings were adopted as a reliable accuracy–cost compromise for all pristine and defect-containing ZnO calculations. Spin–orbit coupling (SOC) was not included in the present calculations; the employed pseudopotentials incorporate scalar-relativistic effects. For wurtzite ZnO, SOC mainly causes a small valence-band splitting and does not alter the defect-induced band-tail trends discussed here; therefore, its omission is not expected to affect the conclusions of this study (Persson and Zunger, 2007).

**TABLE 1:** Convergence tests for k-point mesh and plane-wave cutoff energy (total energy, Ry).

k-point mesh	Total energy (Ry)	Cutoff energy (Ry)	Total energy (Ry)
2 × 2 × 1	−570.79843641	10	−506.86,433,014
3 × 3 × 1	−570.85072701	15	−547.42,047,314
4 × 4 × 1	−570.85686017	20	−563.35691157
5 × 5 × 2	−570.85867407	25	−568.90272810
6 × 6 × 2	−570.86045159	30	−570.50213410
**7 × 7 × 2**	**−570.86092571**	**35**	**−570.83793890**
8 × 8 × 3	−570.86048465	40	−570.88785390
9 × 9 × 3	−570.86037913	45	−570.89355618
10 × 10 × 3	−570.86127927	50	−570.89765010

Bold values indicate the optimized computational parameters (plane-wave cutoff energy of 35 Ry and 7 x 7 x 2 k-point mesh) selected for all subsequent pristine and defective ZnO calculations to achieve a reliable balance between accuracy and computational cost.

The wurtzite ZnO model was constructed by expanding the primitive ZnO unit cell into a 3 × 3 × 1 supercell with lattice parameters a = b = 9.72 Å, c = 5.20 Å and α = β = 90°, γ = 120°. The resulting supercell contains 32 atoms in total (16 Zn and 16 O). After structural relaxation, the electronic band structure and density of states (DOS) were calculated, including both total DOS (TDOS) and partial DOS (PDOS), to clarify the orbital contributions around the valence- and conduction-band edges. From the self-consistent field (SCF) calculations, DOS/PDOS were obtained by projecting the Kohn–Sham states onto Zn and O atomic orbitals using the same relaxed structures and computational settings as above, with Gaussian broadening applied to generate smooth spectra. For the self-consistent field (SCF) cycle, the parameter conv_thr (SCF convergence threshold) was set to 1.0 × 10^−6^ Ry to ensure a well-converged charge density and Kohn–Sham eigenstates for reliable DOS/PDOS analysis of near-edge and defect-related features (Quantum ESPRESSO, 2026).

**FIGURE 4:**
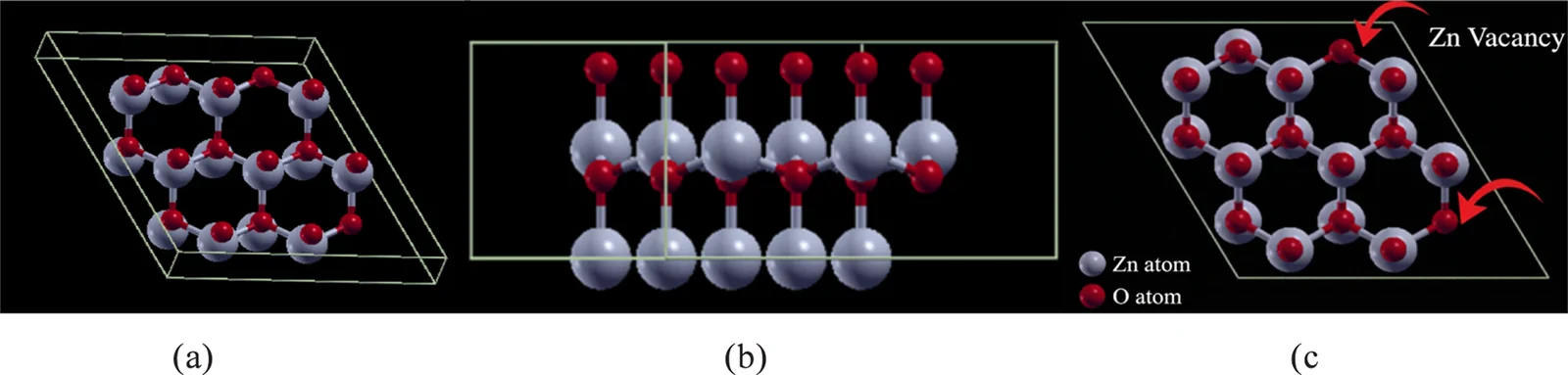
Zinc-deficient ZnO model: **(a)** Perspective view of the ZnO structure with a Zinc-deficient; **(b)** Side view showing the lattice distortion; **(c)** Top view highlighting the vacancy site.

To investigate the influence of intrinsic defects on the electronic structure, a V_Zn_ defect was created by removing one Zn atom from the relaxed 3 × 3 × 1 wurtzite ZnO supercell (32 atoms in total), corresponding to a vacancy concentration of ∼3.1%. The vacancy was introduced at a bulk-like Zn site to minimize finite-size/surface effects. After defect creation, the structure was fully relaxed and the electronic structure (band structure and DOS/PDOS) was recalculated using the same cutoff energy, k-point mesh, and Hubbard U parameters as for pristine ZnO (Figure 4). Zinc vacancies are among the most common intrinsic acceptor-type defects in ZnO and are frequently reported in materials synthesized under low-temperature solution/electrochemical conditions.

## 3 Results and discussion

### 3.1 Characterization of ZnO NRs

**FIGURE 5:**
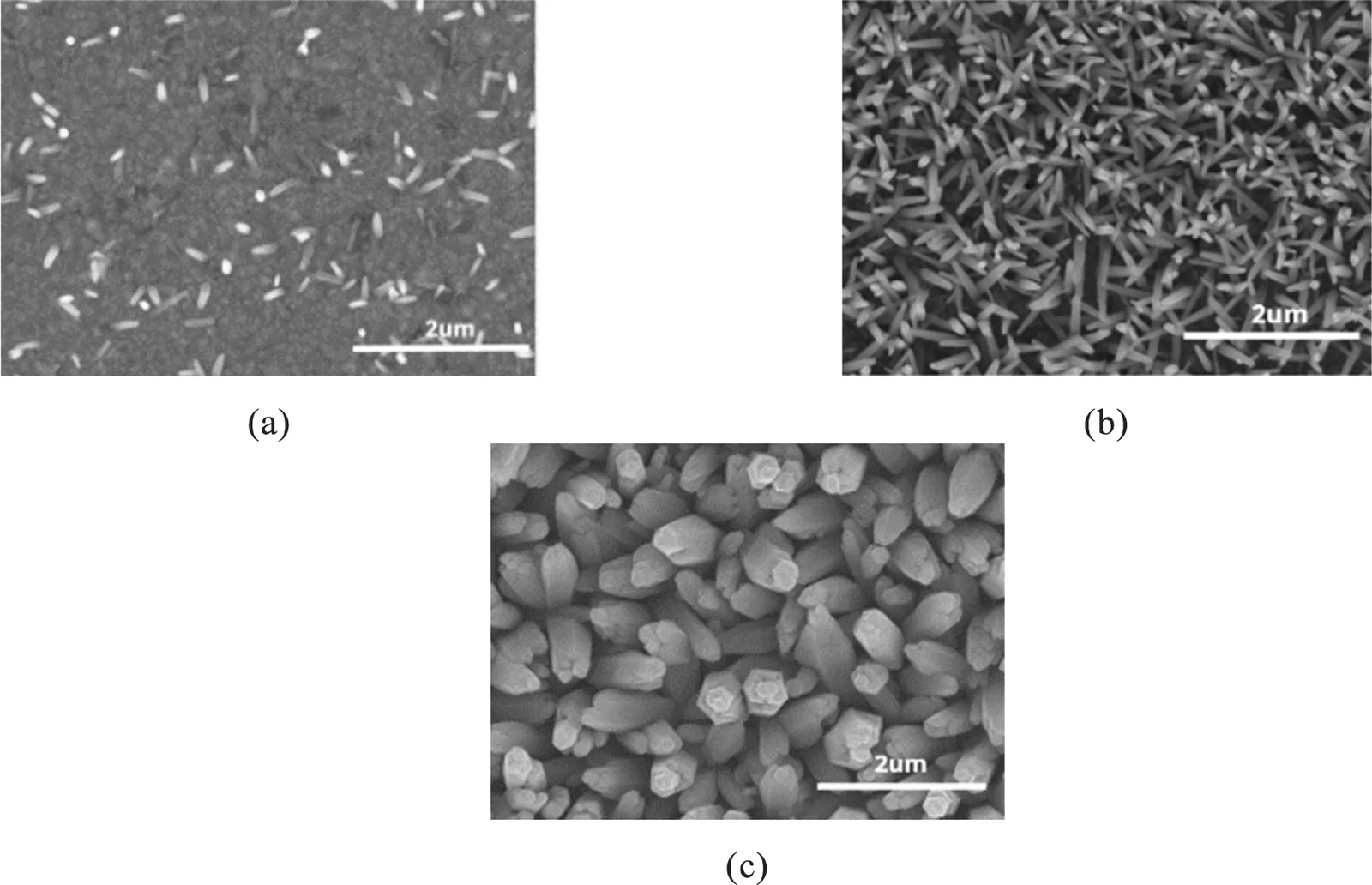
SEM images of ZnO material at different deposition voltages for 30 min at 80 °C: **(a)** 1 V, **(b)** 2 V, and **(c)** 3 V.

Figure 5 shows the SEM images demonstrating the effect of applied potential on the ZnO NRs morphology during the electrodeposition process. The shape and size of ZnO NRs varied with applied voltage. At 1 V (Figure 5a), ZnO NRs were sparse and unevenly distributed on the FTO substrate, indicating slow and inconsistent growth. Increasing the voltage to 2 V resulted in denser (Figure 5b), longer ZnO NRs, forming a coating on the surface. At 3 V (Figure 5c), ZnO NRs grew at the highest density, covering the entire substrate. The hexagonal wurtzite structure of ZnO NRs was clearly visible at 2 V and 3 V. However, while higher nanorod density can be advantageous in various photoelectric devices due to increased surface area and light harvesting, the optimal density depends on the target application. In the present PEC glucose-sensing configuration, an excessively dense ZnO NR layer can reduce effective light penetration into the electrode, hinder electrolyte access/ion diffusion within the nanorod forest, and increase charge recombination at nanorod–nanorod junctions, thereby lowering the effective photocurrent response (Dhara and Mahapatra, 2017; Li et al., 2019; Wang et al., 2019; Guler et al., 2023). Therefore, 2 V was determined to be the optimal voltage for producing ZnO NRs under these experimental conditions.

**FIGURE 6:**
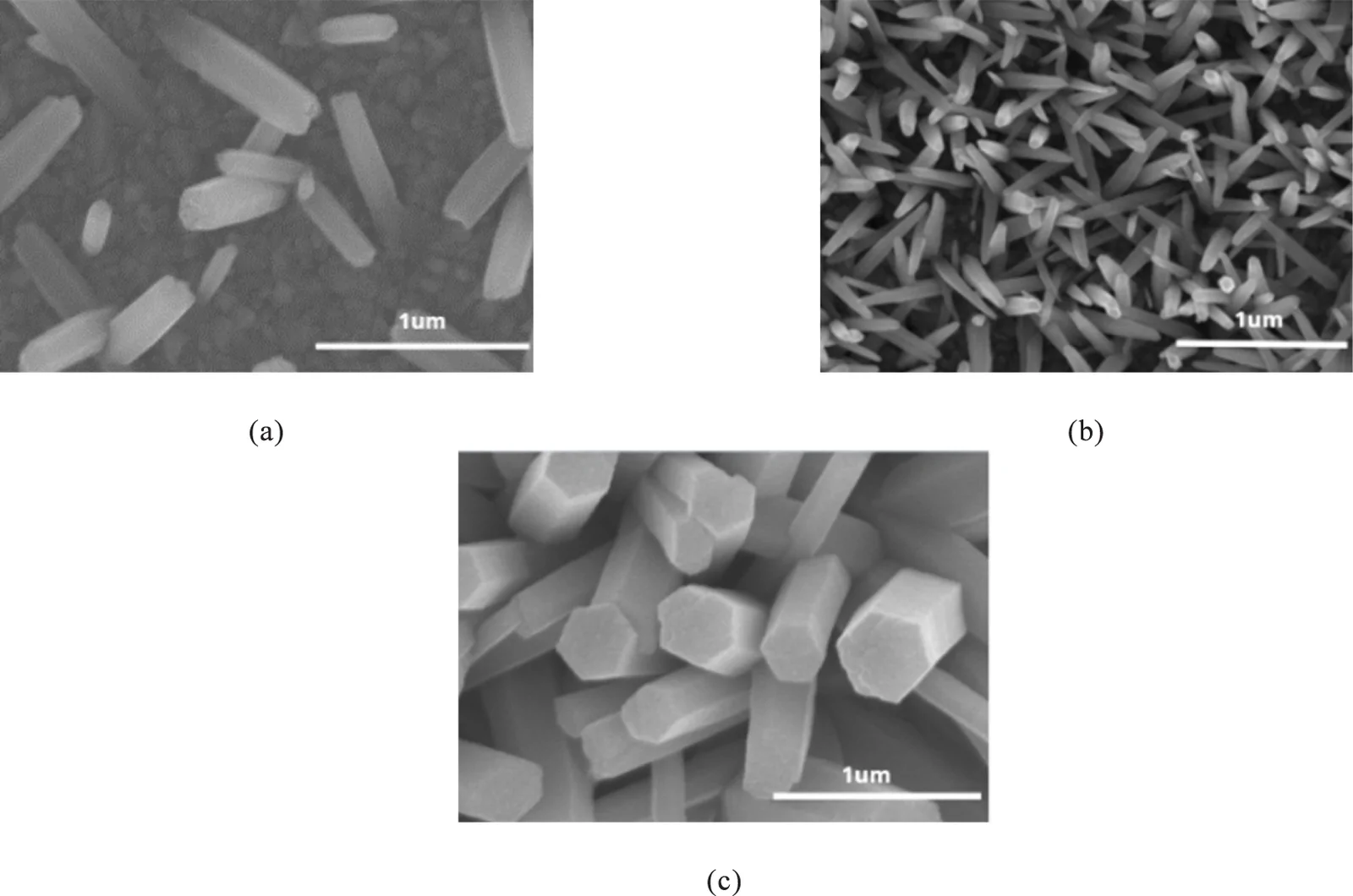
SEM images of ZnO material on FTO substrate at a deposition voltage of 2 V and 80 °C for different synthesis durations: **(a)** 20 min; **(b)** 30 min; **(c)** 40 min.

Figure 6 demonstrates the influence of synthesis duration on ZnO NRs morphology. Electrodeposition for 20 min yielded small, uneven, and sparsely distributed ZnO NRs (Figure 6a). Increasing the deposition time to 30 min resulted in larger, more numerous ZnO NRs, with a visible but less defined hexagonal structure (Figure 6b). At 40 min, ZnO NRs reached their maximum size, and the hexagonal structure became well-defined (Figure 6c). While longer synthesis times generally produced better-developed NRs, 40 min resulted in ZnO NRs with a diameter of approximately 470 nm, exceeding the size typically aimed for in high-surface-area applications. The synthesis temperature of 80 °C and prolonged duration contributed to solution evaporation and uneven material deposition on the FTO substrate. Therefore, 30 min was identified as the optimal synthesis time under these experimental conditions.

**FIGURE 7:**
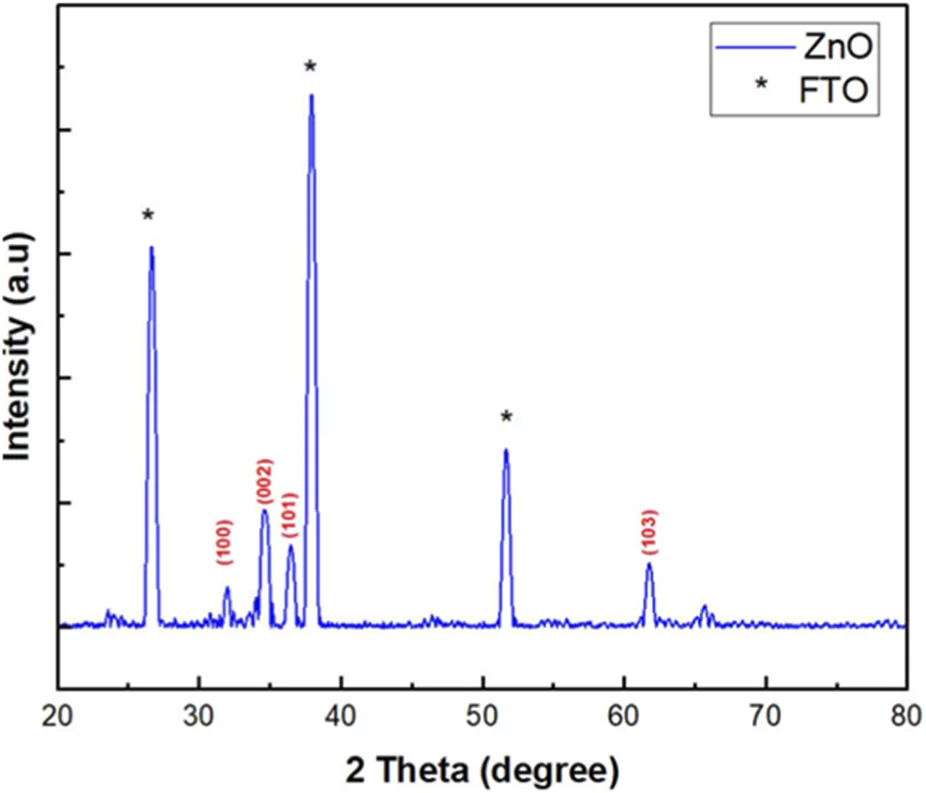
XRD pattern of ZnO NRs/FTO.

XRD analysis (Figure 7) revealed diffraction peaks at 32.29°, 34.76°, 36.75°, and 62.09°, corresponding to the (100), (002), (101), and (103) planes of hexagonal wurtzite ZnO. The prominent (002) peak indicated preferential growth along the c-axis, typical of ZnO NRs (NRs). The FTO substrate exhibited diffraction peaks at approximately 26.5°, 37.8°, and 51.7°, characteristic of the cassiterite structure with fluorine doping. These peak positions may vary slightly due to changes in electron density and synthesis conditions. Overall, the XRD results confirmed the successful synthesis of ZnO NRs on the FTO substrate.

**FIGURE 8:**
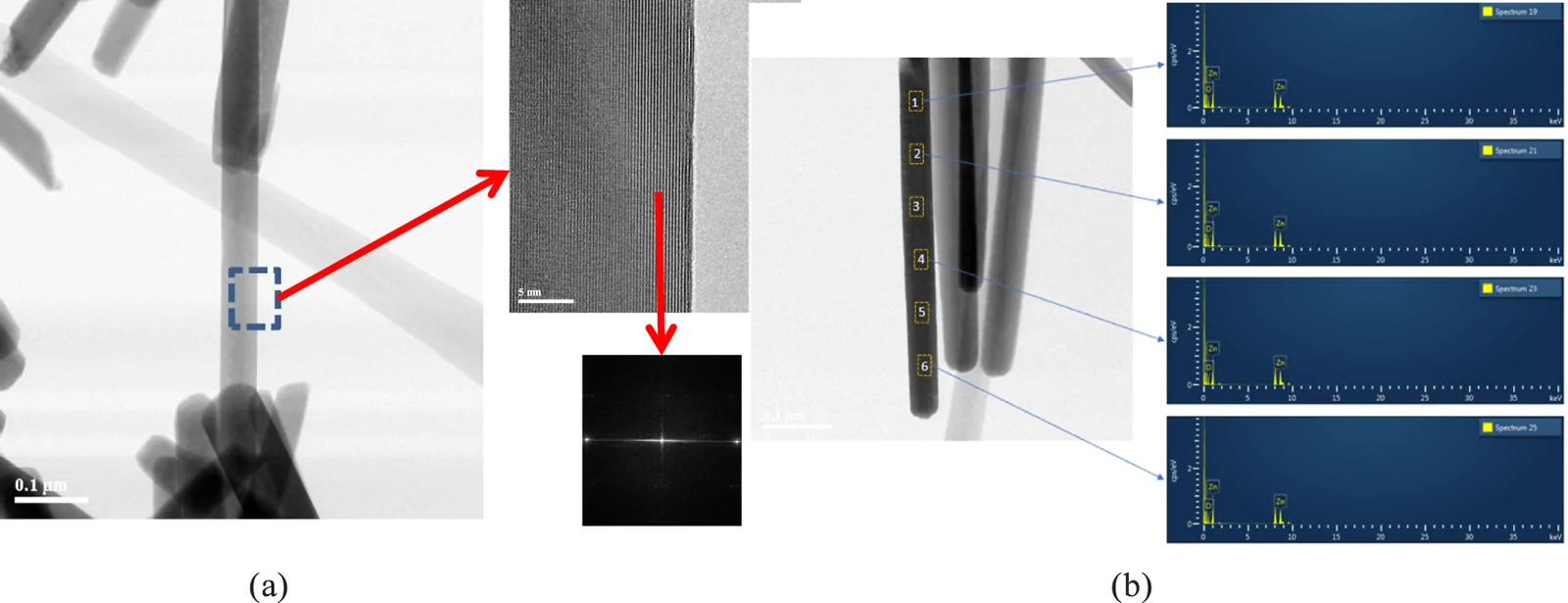
HRTEM **(a)** and EDX **(b)** characterization of ZnO NRs/FTO.

To further support the structural analysis, HRTEM/EDX (Figure 8) measurements were carried out on individual ZnO nanorods at multiple positions along the rod (positions 1–6 from base to tip). The EDX quantification reveals a noticeable deviation from the ideal 1:1 stoichiometry (Table 2), with the oxygen content increasing from **49.60% to 56.91%** while the zinc content correspondingly decreases from **50.40% to 43.09%** when moving from the root region toward the tip.

**TABLE 2:** Atomic percent of O and Zn with different position in ZnO NR.

​	Position 1 (Base)	Position 2	Position 3	Position 4	Position 5	Position 6 (Tip)
O (at%)	49.6	48.56	50.68	52.26	54.88	56.91
Zn (at%)	50.4	51.44	49.32	47.74	45.12	43.09
Total	100	100	100	100	100	100

Positions 1 to 6 correspond to the sequential measurement locations along the individual ZnO nanorod analyzed via EDX in Figure 8.

This spatially varying Zn/O ratio (Zn/O < 1.0) confirms a Zn-deficient environment, which is thermodynamically favorable for the spontaneous formation of zinc vacancies V_Zn_ under the O-rich, low-temperature electrodeposition conditions ^[13, 14]^. This compositional gradient can be rationalized by considering the role of the ZnO seeded layer and the growth/feeding pathway during electrodeposition. Near the nanorod base, the crystal is in direct contact with the underlying ZnO seed layer, which can facilitate local Zn supply (i.e., act as a Zn reservoir) and provide diffusion pathways for Zn-related species at the rod/substrate interface (Yoo et al., 2014). As the growth front advances away from the substrate, the effective Zn supply to the upper segments becomes increasingly limited by diffusion length and mass transport, while a higher density of defect sites can trap Zn species or favor Zn-deficient incorporation. Consequently, the upper region tends to be more Zn-deficient than the base, providing direct evidence for non-uniform V_Zn_ formation and aligning with the defect-mediated band-gap narrowing captured by our DFT + U model.

**FIGURE 9:**
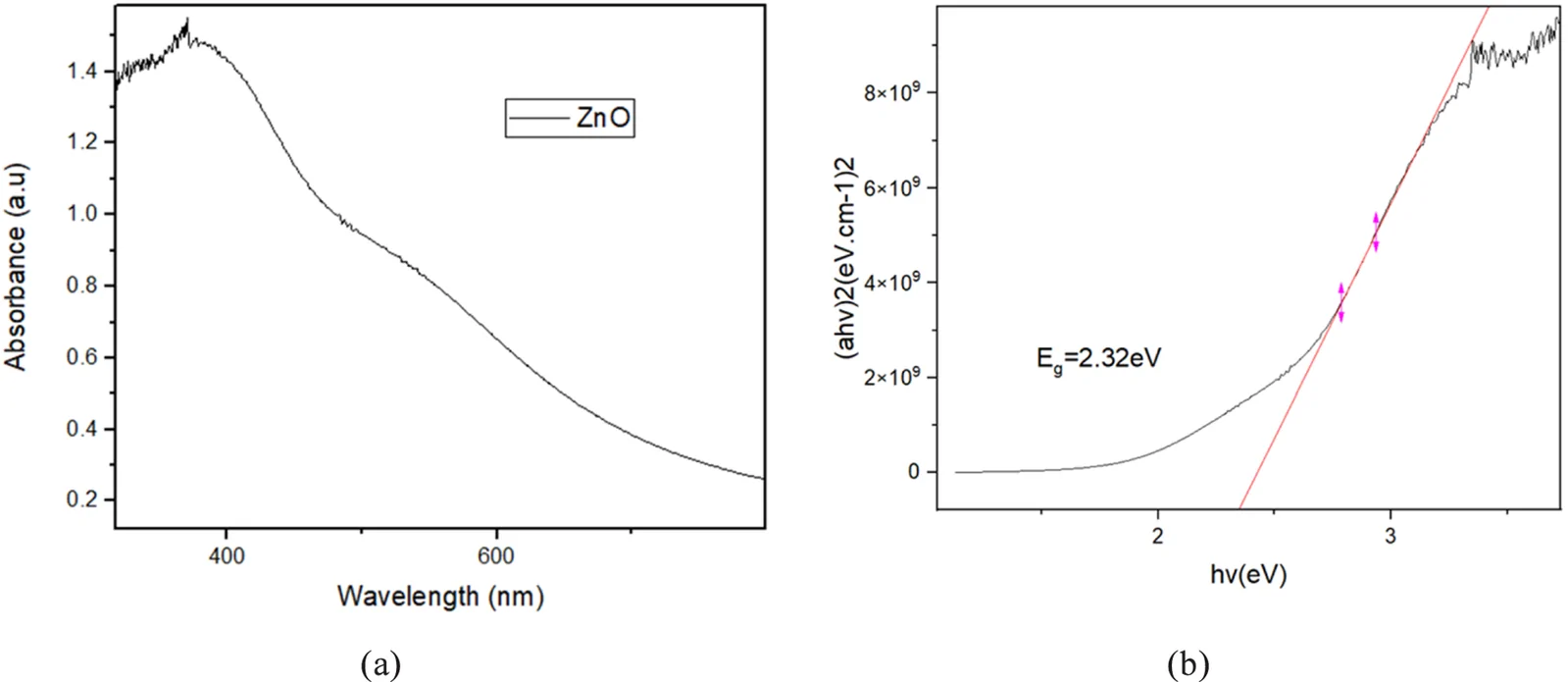
UV–Vis absorption spectra **(a)** and band gap calculation (Tauc plot) **(b)** diagram for ZnO NRs/FTO.

Figure 9A shows the UV-Vis absorption spectrum of ZnO NRs/FTO reveals an absorption peak at 420 nm, suggesting suitable excitation wavelengths for PEC measurements. Using the conventional Tauc plot approach (Figure 9B), the optical gap of the synthesized ZnO NRs/FTO sample is estimated to be 2.32 eV. It should be noted that for defect-rich ZnO, the presence of band-tail (Urbach) states and additional sub-bandgap absorption can bias the linear extrapolation in Tauc analysis and may lead to an apparent optical gap smaller than the intrinsic band gap of stoichiometric ZnO (∼3.3 eV); therefore, the value reported here should be interpreted as an effective/apparent optical gap associated with defect-assisted optical transitions rather than a pristine band-edge transition (Yang et al., 2017; Ye et al., 2021). In this case, the defect-related nature of this reduced optical gap is supported by converging structural, compositional, and theoretical evidence: XRD (Figure 7) confirms the wurtzite ZnO phase on FTO without detectable impurity phases within the instrument resolution, spatially resolved EDX (Figure 8; Table 2) reveals a Zn-deficient composition consistent with intrinsic point defects, and DFT + U calculations predict defect-induced band-tail/near-edge states that can enable visible-light excitation (Section 3.4). While the presence of minor amorphous impurities cannot be completely ruled out by XRD alone, the combined structural, compositional, and theoretical evidence supports defect-mediated sub-bandgap absorption as the dominant origin of the observed visible-light photoresponse. ZnO nanocrystals can exhibit various defects, such as Zn and O vacancies, interstitials, and antisite defects, which often arise during low-temperature growth. These defects introduce intermediate energy levels within the bandgap, reducing its width. While this may lead to shorter electron-hole pair lifetimes, it does not pose a significant problem in PEC applications due to electrode biasing. The applied electric field effectively drives electron-hole separation, facilitating their participation in glucose oxidation reactions before recombination.

### 3.2 Proposed mechanism of ZnO NRs growth via electrodeposition

In the initial experimental design, SEM was used as a rapid morphology-screening step to identify deposition conditions that yield mechanically robust and well-aligned ZnO nanorods for reproducible sensing. Here, “mechanical stability” was assessed operationally based on the integrity of the ZnO NR layer after sample rinsing/drying and routine handling (i.e., no visible peeling, cracking, or large-area delamination), while “alignment” was judged from SEM morphology by the prevalence of vertically oriented rods (limited tilting/bundling) and uniform surface coverage with minimal large voids or agglomerates (Ghannam et al., 2018; Zheng et al., 2013). At 1 V, ZnO nucleation/growth was insufficient, producing a sparse and discontinuous layer that is unfavorable for charge transport. At 3 V, the deposition rate was excessively high, resulting in coarse and non-uniform nanorods with reduced adhesion to FTO. Therefore, the sample deposited at 2 V for 30 min was selected for further characterization (XRD, UV–Vis, PEC, and DFT + U) due to its superior structural integrity and optimal c-axis orientation.

Through the process in Figure 1, the growth of ZnO NRs via the electrodeposition method can be explained as follows. The specific reactions are as follows:

$${\left({\text{CH}}_{2}\right)}_{6}N_{4}+6H_{2}O\to 6\text{HCHO}+4NH_{3}$$ (1)

$${\text{NH}}_{3}+H_{2}O\;⇌\;{\text{NH}}_{4}^{+}+{\text{OH}}^{-}$$ (2)

$${\text{Zn}}^{2+}+2OH^{-}\Leftrightarrow \text{ Zn}{\left(\text{OH}\right)}_{2}$$ (3)

$$\text{Zn}{\left(\text{OH}\right)}_{2}\to \text{ZnO}+H_{2}O$$ (4)

The structural anisotropy of the wurtzite lattice plays a key role in governing the spatial distribution of these intrinsic defects. Wurtzite ZnO crystals possess distinct polar top faces namely the Zn-terminated $\left(0001\right)$ or O-terminated $\left(000\overset{ˉ}{1}\right)$ surfaces and non-polar side-walls such as the $\left\{10\overset{ˉ}{1}0\right\}$ m-planes. Because polar surfaces exhibit substantially higher surface energy than non-polar facets, they serve as the primary driving force for rapid axial growth, which is heavily reflected in the dominant $\left(002\right)$ diffraction peak in our XRD pattern (Figure 7). Under the low-temperature, kinetically controlled electrodeposition conditions used here, the advancing polar growth front at the nanorod tip experiences local mass-transport limitations and structural stress. This high-energy polar surface significantly lowers the thermodynamic formation energy of cation vacancies, actively favoring the spontaneous incorporation of $V_{Zn}$ over stable non-polar side-wall incorporation. Consequently, this atomic-scale surface activity rationalizes the progressively lower Zn/O ratio observed toward the tip region (Table 2), establishing the $V_{Zn}$ -rich polar tip as a highly active electronic and catalytic site that directly enables visible-light photoelectrochemical glucose oxidation.

Upon dissolving Zn(NO_3_)_2_.6H_2_O in distilled water, complete dissociation into Zn^2+^ and NO^3-^ ions occurs. Heating initiates the hydrolysis of C_6_H_12_N_4_, yielding ammonia (NH_3_) and formaldehyde (HCHO) (Equation 1). As a weak base, NH_3_ accepts a proton from water to form NH_4_^+^ and OH^−^ (Equation 2). The Zn^2+^ ions then react with OH^−^ to produce Zn(OH)_2_ precipitate (Equation 3). However, Zn(OH)_2_ is unstable at high temperatures and continued heating leads to the dehydration of Zn(OH)_2_, resulting in the formation of ZnO (Equation 4). When a voltage is applied between the anode and cathode, a current flows through the solution, causing Zn^2+^ ions to migrate towards the cathode. Upon encountering OH^−^ ions at the substrate surface, Zn^2+^ and OH^−^ ions react to form Zn(OH)_2_ precipitates, which subsequently decompose into ZnO, adhering firmly to the surface.

### 3.3 Mechanism of glucose detection using ZnO NRs with EC and PEC measurements

In EC glucose sensors, ZnO-coated electrodes facilitate the oxidation of glucose in an alkaline environment, such as NaOH, producing gluconolactone, electrons, and water. The generated electrons contribute to the current signal, represented by the reaction in Equation 5 (Dong et al., 2021):

$$\text{Glucose}+{\text{OH}}^{‐}\;\to \text{ Gluconolactone}+e^{‐}+H_{2}O$$ (5)

In PEC sensors, when ZnO NRs are exposed to Light excitation, they generate electron-hole pairs, in which the holes (h^+^) react with water molecules to form reactive hydroxyl radicals (OH^•^). These radicals then participate in the oxidation of glucose, enhancing the reaction efficiency. The main reactions in this process are described in Equations 6-8 (Dong et al., 2021):

$$\text{ZnO}+h\nu \;\to \;h^{+}\left(\text{ZnO}\right)+e^{‐}\left(\text{ZnO}\right)$$ (6)

$$h^{+}\left(\text{ZnO}\right)+H_{2}O\;\to \;{\text{OH}}^{\cdot }\left(\text{ZnO}\right)$$ (7)

$$\text{OH}\cdot \left(\text{ZnO}\right)+\text{Glucose }\to \text{ Gluconolactone}+\text{Intermediate products}+e^{‐}$$ (8)

### 3.4 Electrochemical and photoelectrochemical response with glucose of ZnO NRs

**FIGURE 10:**
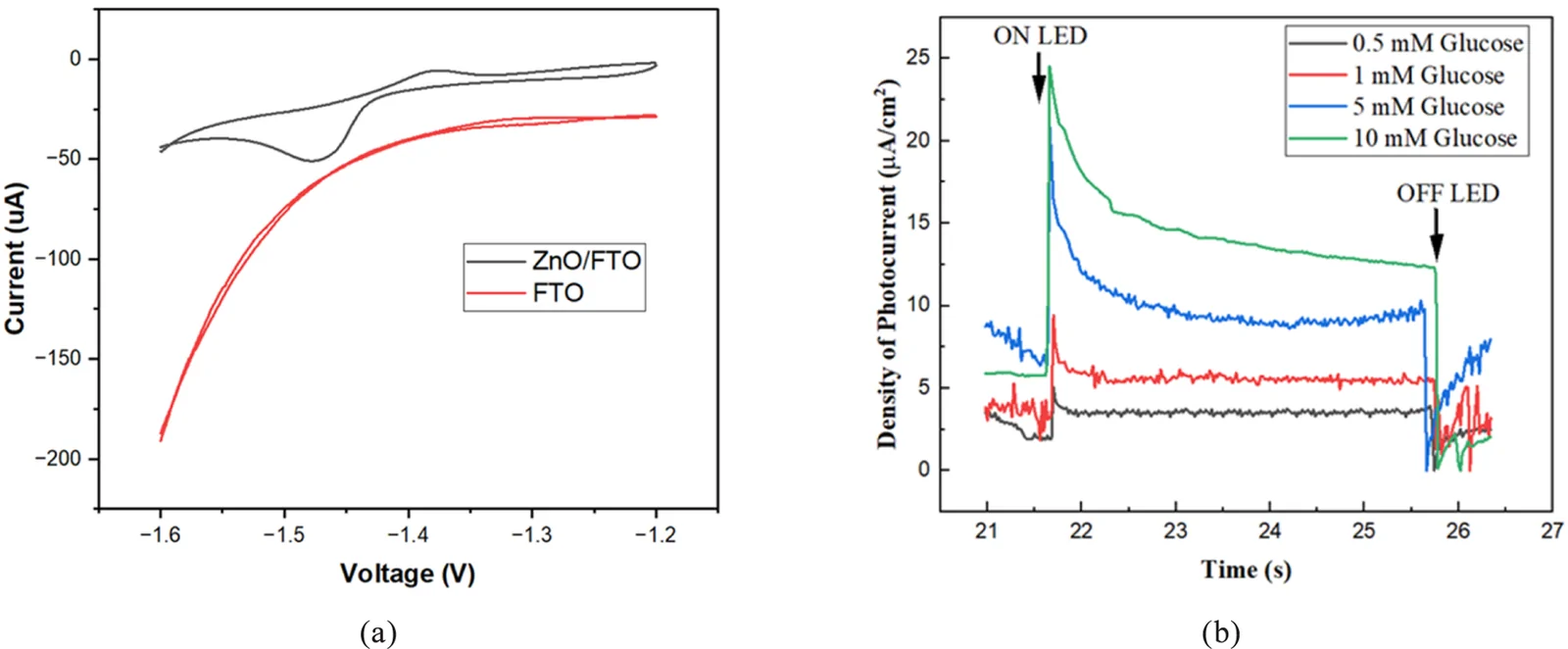
Electrochemical and Photoelectrochemical Response of ZnO NRs/FTO with glucose: **(a)** CV curves of FTO, and ZnO NRs/FTO with 3 mM glucose; **(b)** Photochronoamperometry of ZnO NRs/FTO at different concentrations when on/off LED 460 nm.

**FIGURE 11:**
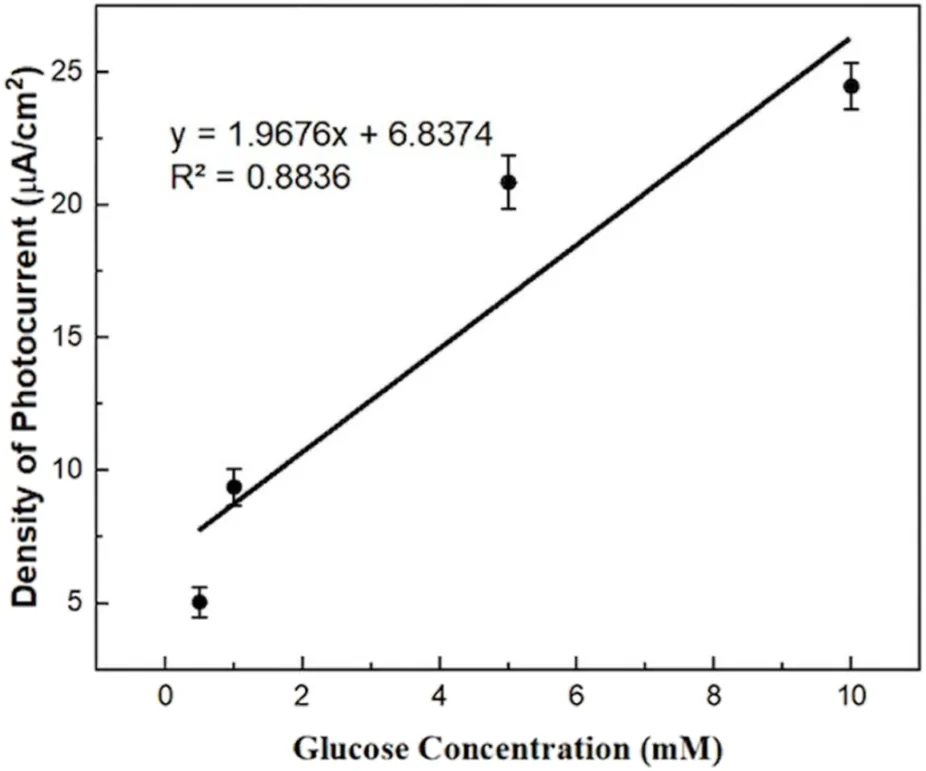
Linear calibration curve of the ZnO NRs/FTO electrode for PEC glucose detection. Error bars represent the standard deviation of three independent measurements.

The CV measurements (Figure 10A) revealed a significant difference in the electrochemical signal between FTO substrates with and without ZnO NRs, indicating enhanced electrochemical activity in the presence of ZnO NRs under the present measurement conditions. The CV curves showed prominent current features at approximately −1.35 V and −1.45 V (vs. Ag/AgCl) within the applied potential window; however, these features are not assigned here as unambiguous glucose redox peaks because their positions can vary substantially with electrolyte composition/pH, scan window, and electrode polarization/overpotential, and literature reports often observe glucose-related peaks at less negative potentials for ZnO-based electrodes (Zhou et al., 2022). In general, glucose-related peaks observed at less negative potentials are commonly associated with lower overpotential and more favorable reaction kinetics, whereas the more negative features observed here may reflect stronger electrode polarization under the selected scan window and electrolyte conditions (Zhou et al., 2022). In this work, CV was used primarily to identify a practical bias region for subsequent photochronoamperometry, whereas the glucose-dependent photocurrent response under 460 nm illumination (Figures 10B, 11) provides the direct evidence for glucose sensing.

Figure 10b shows the photochronoamperometry of ZnO NRs when they were irradiated with the excitation light of 460 nm at different concentrations. The PEC signal exhibited a clear response to glucose concentrations of 0.5 mM, 1 mM, 5 mM, and 10 mM, indicating that changes in glucose concentration translate into a measurable photocurrent variation under visible-light illumination. To quantify this experimentally observed response, the sensitivity was defined as the slope (ΔJ/ΔC) of the calibration curve relating the steady-state photocurrent density (J) to glucose concentration (C) at the fixed applied bias. However, a rapid decrease in current density over time was observed, consistent with the known rapid electron-hole recombination of ZnO NRs. The linear relationship between photocurrent density (J) and glucose concentration (C) for ZnO NRs/FTO is shown in Figure 11, with a correlation coefficient (R^2^) of 0.88. Although this R^2^ value is lower than ideal for sensor calibration, it is plausibly influenced by time-dependent photocurrent decay caused by carrier recombination and interfacial instability; in future work, improving nanorod/substrate adhesion and optimizing/controlling the defect concentration (to balance visible-light absorption and recombination) are expected to mitigate signal drift and further enhance the linearity. The equation for this relationship is: J (μA/cm^2^) = 1.9676 x C (mM) + 6.8374. Using the formula LOD = 3σ/s, where σ is the standard deviation of the blank signal and s is the sensitivity (Dhara and Mahapatra, 2017), the limit of detection (LOD) was calculated to be 12.5 μM. Table 3 shows the comparison of the PEC performance of the ZnO NRs/FTO electrodes with other glucose-sensing systems. The present system demonstrated a low detection limit, high sensitivity (0.5–10 mM), and competitive overall performance.

**TABLE 3:** Comparison of linear range and LOD of various EC/ PEC sensors for non-enzyme glucose testing.

Material	Method	Linear range	LOD
ZnO NRs/FTO (this work)	PEC	0.5 mM – 10 mM	12.5 μM
Ti_3_C_2_/Cu_2_O (Li et al., 2019)	PEC	0.5 nM – 0.5 mM	0.17 nM
Fe_2_O_3_ Rs/FTO (He et al., 2020)	PEC	0.2 – 2 mM	5.5 μM
CdS QDs (Mao and Zhang, 2022)	PEC	0.05 – 1,000 μM	15.99 nM
Ni(OH)_2_/TiO_2_ (Yang et al., 2017)	PEC	0.5 – 20 mM	0.23 μM
Au NPs/ZnO (Ye et al., 2021)	PEC	1 μM – 6.917 mM	0.1 μM
Au/Pt/Nf (Chinnadayyala et al., 2018)	EC	1 – 40 mM	23 μM
Au-Ru NPs (Nguyen et al., 2020)	EC	1 – 10 mM	0.068 mM
PtPd/GCE (Bo et al., 2011)	EC	1 – 2.5 mM	0.12 mM

The operational parameters of the V_Zn_ ZnO NRs/FTO platform were further rationalized. The repeatability and reproducibility of the sensor are reflected in the small standard deviations shown in the error bars of Figure 11, stemming from consistent batch-to-batch electrodeposition. In terms of stability, since this platform is conceptualized as a disposable, single-use PEC diagnostic strip (in line with commercial blood glucose testing standards), long-term continuous reuse is not required. The device exhibits excellent short-term operational stability, as evidenced by the stable steady-state photocurrent plateaus reached in Figure 10B. Furthermore, the selectivity of the sensor under 460 nm visible light at pH 7.4 is favored by the low operating bias, where the oxidation of common biological interferents like ascorbic acid and uric acid is kinetically blocked compared to the highly efficient, defect-mediated catalytic oxidation of glucose, consistent with recent advancements in metal oxide nanostructures for non-enzymatic diabetes management (Naikoo et al., 2021).

It is worth noting that while the present study demonstrates the viable potential of Zn-deficient ZnO NRs for visible-light-driven glucose detection, a rigorous validation of the comprehensive electrochemical mechanism—including detailed experimental profiles of boundary-layer mass transfer modes, extended selectivity matrices against complex co-interferents, and real-sample matrix effects—remains to be fully established. These multi-physical sensing parameters are designated for dedicated future investigations utilizing this point-defect engineered ZnO platform.

### 3.5 Electronic structure from DFT + U

**FIGURE 12:**
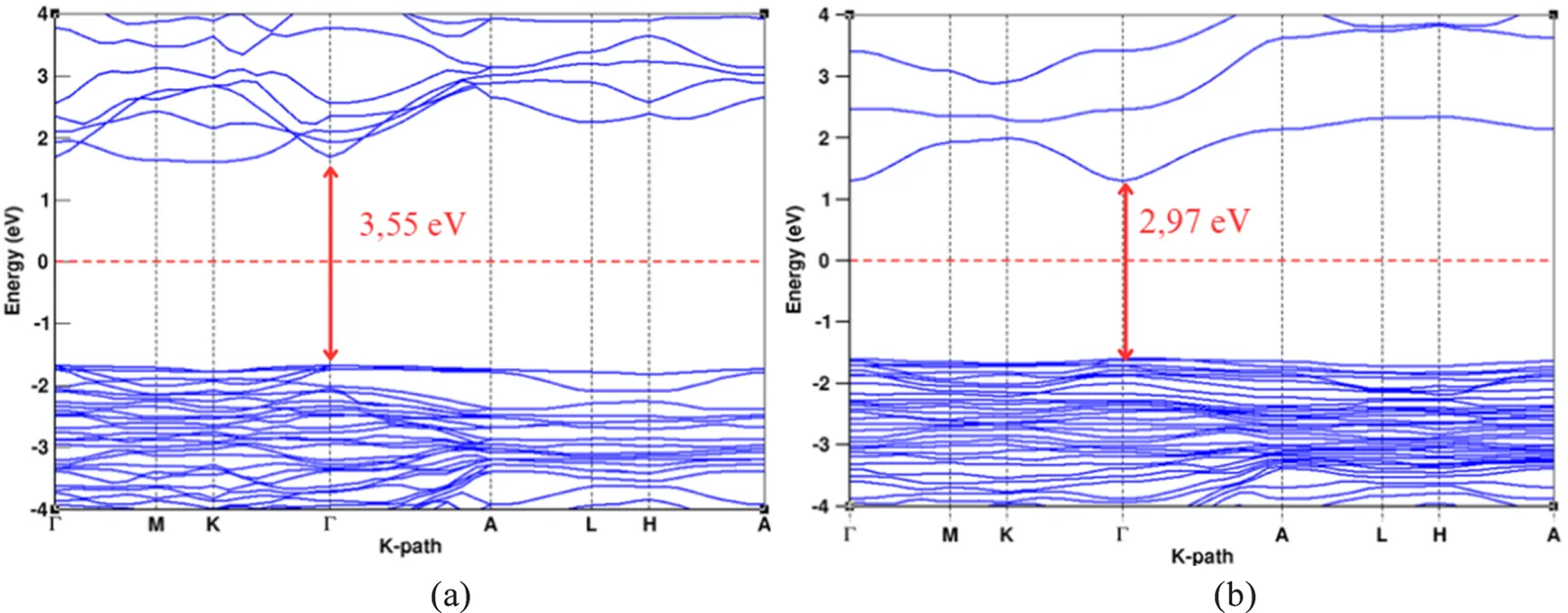
Energy band gap analysis of: **(a)** pristine ZnO and **(b)** V_Zn_-related defects ZnO.

Figure 12 shows that introducing a V_Zn_ into the ZnO model modifies the electronic structure by introducing vacancy-related defect states near the band edges and effectively narrowing the bandgap. As extracted from Figures 12A,B, the calculated band gap decreases from **3.55 eV** for pristine ZnO to **2.97 eV** for the V_Zn_-defective model, consistent with the defect-assisted visible-light activation discussed above. This theoretical trend is consistent with the experimental UV–Vis/Tauc analysis (Figure 9), which yields an optical bandgap of **E**_**g**_ **≈ 2.32 eV** for the electrodeposited ZnO nanorods. In other words, sub-bandgap optical transitions in defect-rich ZnO can generally arise from multiple intrinsic defects, including zinc vacancies (V_Zn_), oxygen vacancies (V_O_), and zinc interstitials (Z_i_) (Janotti and Van de Walle, 2007; Lany and Zunger, 2010; Oba et al., 2008). In the present work, while contributions from other defects cannot be completely excluded, the Zn-deficient composition revealed by spatially resolved EDX and the DFT + U results support V_Zn_-related band-tail/near-edge states as the primary contributors enabling photoexcitation under 460 nm illumination (photon energy ∼2.7 eV), which is not possible for ideal ZnO (E_g_ ∼3.3 eV).

In this work, defect-related electronic states were probed using a combination of experimental and computational approaches. Experimentally, UV–Vis absorption (Tauc analysis, Figure 9) provides evidence of band-gap narrowing/sub-bandgap absorption, while spatially resolved EDX (Figure 8; Table 2) supports a Zn-deficient composition consistent with V_Zn_-type defects. Computationally, DFT + U electronic-structure calculations (band structure and DOS/PDOS) were used to identify defect-induced band-tail/near-edge states. Although photoluminescence (PL) spectroscopy and deep-level transient spectroscopy (DLTS) are established techniques for resolving defect levels in ZnO, they were not performed in the present study (Özgür et al., 2005).

Compared with pristine ZnO, the Zn-deficient model exhibits the emergence of defect-induced states (often described as mid-gap/band-tail states) associated with V_Zn_. These states effectively reduce the energy required for electron excitation and promote carrier generation under visible-light irradiation, thereby supporting the observed PEC activity of the electrodeposited ZnO nanorods.

The DOS/PDOS analysis further clarifies the experimental mechanism. In ZnO, the valence band is dominated by O-2p states, whereas the conduction band is mainly contributed by Zn-4s states with hybridization from Zn-3d. When a cation-vacancy defect such as V-Zn is present, the redistribution of electronic states near the band edges can introduce vacancy-related levels that act as stepping stones for excitation and transport, improving the probability that photogenerated electrons are collected through the FTO while photogenerated holes participate in interfacial oxidation reactions.

Under 460 nm illumination, the presence of V_Zn_-induced defect states facilitates visible-light absorption, enhances carrier generation, and promotes efficient charge separation and interfacial charge transfer, resulting in a glucose-dependent photocurrent. In this context, the DFT + U results provide a coherent electronic-structure framework that rationalizes the experimentally observed visible-light-driven PEC glucose sensing behavior of the electrodeposited ZnO nanorods and highlights the critical role of V_Zn_-related defect states in tailoring the electronic properties of ZnO.

From the band structure of both the pristine and zinc-deficient models, it can be observed that in the zinc -deficient structure, conduction band tail states may arise due to electron disorder. The conduction band tail may extend below the conduction band minimum or shift upward toward the maximum of the valence band. Electrons associated with lattice disorder in the crystal structure can generate new electronic states near the conduction band minimum or shift toward the valence band edge of the crystal. The main effect of modifying the nanomaterial structure is the formation of strong band-tail states near the conduction band edge, which leads to a reduction in the bandgap.

**FIGURE 13:**
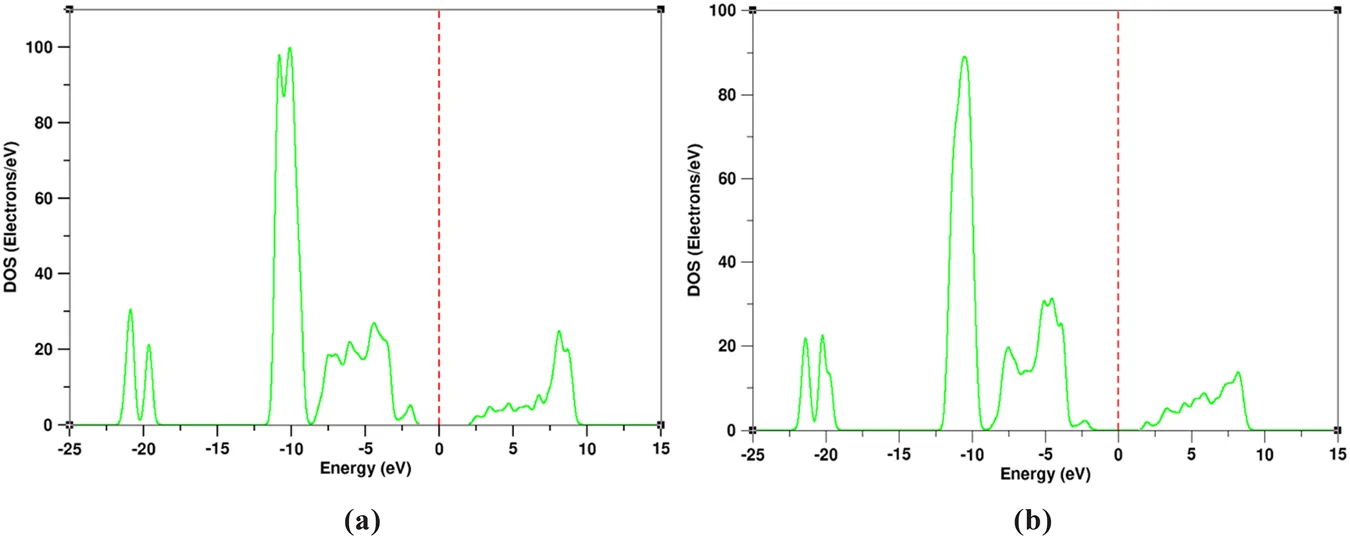
The total density of states of the ZnO model **(a)** and zinc-deficient ZnO model **(b)**.

**FIGURE 14:**
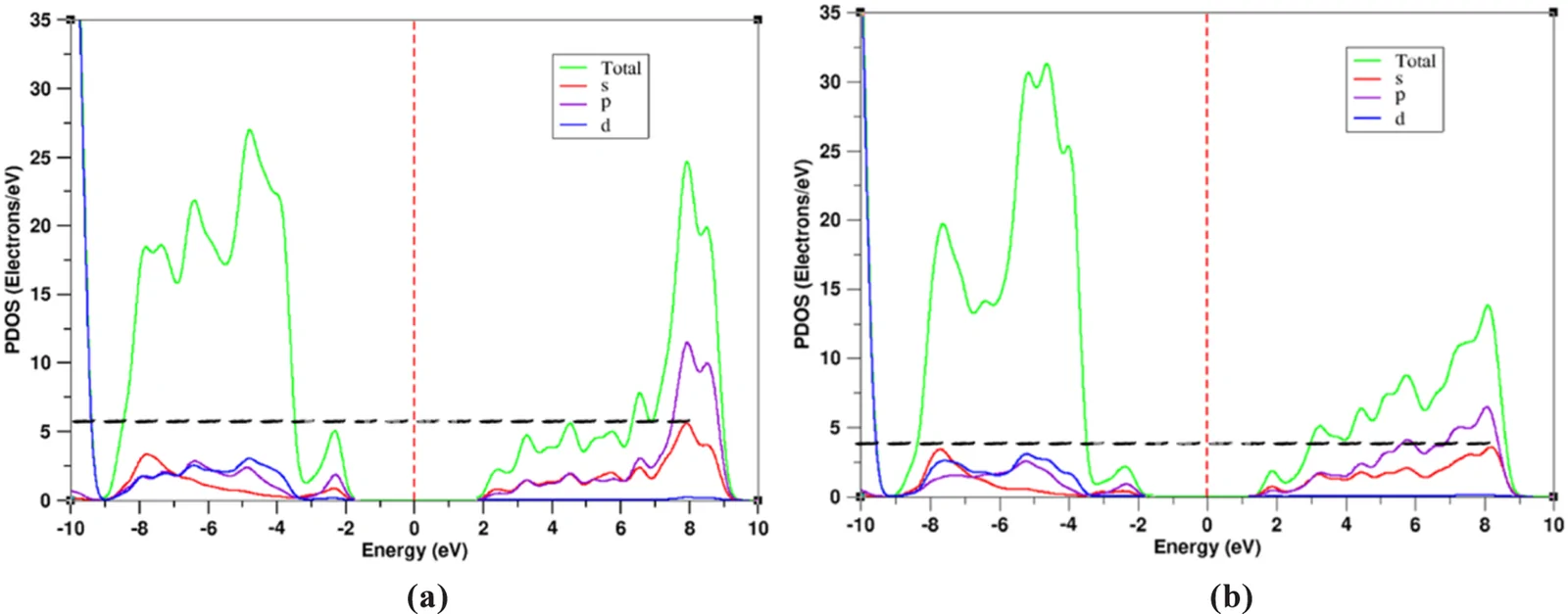
The PDOS of Zn in ZnO model **(a)** and zinc-deficient ZnO model **(b)**.

Physically, this band-edge/DOS narrowing reflects the formation of vacancy-induced localized (band-tail) states near the valence- and conduction-band edges (see Figure 12 for the band-structure-derived band-gap narrowing; Figure 13 for the corresponding TDOS change; Figure 14 for the Zn-projected PDOS signature). These states reduce the effective excitation energy (i.e., apparent band-gap reduction) and extend optical absorption into the visible range (Figure 9), thereby increasing the probability of generating photocarriers under 460 nm illumination and facilitating interfacial charge transfer during glucose oxidation, which is consistent with the enhanced PEC response observed in Figures 10B, 11.

Therefore, the maximum wavelength of the excitation radiation that ZnO can absorb is estimated using Equation 9:

λmax=hcEg=6.625×10‐34×3×1082.97×1.6×10‐19=418 nm (9)

Using the DFT + U bandgap values extracted from Figure 12, E_g_ decreases from 3.55 eV (pristine ZnO) to 2.97 eV (V_Zn_-defective ZnO), shifting λ_max_ to longer wavelengths and supporting defect-assisted photoexcitation in the visible range. Therefore, the observed DOS/band-edge changes provide a direct electronic-structure basis for enhanced photocarrier.

From an overall perspective, the total density of states (DOS) of zinc-deficient ZnO shows a significant change (Figure 13). The **electronic energy range** of the model is narrowed from **[−12.3, +10] eV** (Figure 13.a) to **[−12.5, +10] eV** (Figure 13.b) upon the introduction of zinc vacancies. The energy levels in this range correspond to the upper states of the valence band and the lower states of the conduction band.

The pronounced band-edge modification observed in Figure 13 can be interpreted more directly from the PDOS in Figure 14 by considering the electronic configuration of Zn. In pristine ZnO, the valence band is mainly composed of O-2p states, whereas the conduction band is dominated by Zn-4s states. Zinc has the configuration [Ar]3d^10^4s^2^; therefore, creating a V_Zn_ effectively removes the Zn-4s electrons from the lattice and leaves a hole-like (acceptor) center associated with the missing 4s-derived states. Consistent with this picture, the Zn-related contribution in the conduction-band region is strongly reduced in the zinc-deficient model, with the PDOS peak intensity decreasing from about 6 electrons/eV (Figure 14A) to 3.75 electrons/eV (Figure 14B). The resulting hole can attract and trap free electrons, while simultaneously introducing localized defect states within (or near) the band gap that act as intermediate transition levels. Consequently, visible photons can first promote electrons from the valence band to these vacancy-related states and then further to the conduction band, producing additional electron–hole pairs and forming band-tail states near the band edges. This defect-assisted pathway shifts the effective band edges toward the Fermi level, narrows the apparent band gap, and extends the absorption edge into the visible region, thereby enhancing the visible-light photoresponse of ZnO.

These PDOS results confirm that the electronic-structure changes are governed by the concentration of zinc vacancies: the vacancy perturbs the Zn sub-lattice and generates vacancy-related localized states near the band edges, while the overall Zn spectral features away from the band edge remain comparatively unchanged. Therefore, the variation in peak intensity around the band edges in Figure 13 can be used as a qualitative indicator of V-Zn-induced defect density and its role in band-gap narrowing and visible-light absorption.

Therefore, the variation in peak intensity around the band edges in Figure 13 can be used as a qualitative indicator of V_Zn_ induced defect density and its role in band-gap narrowing and visible-light absorption. The observed visible-light absorption (extending to ∼460 nm) and the bandgap narrowing identified in the Tauc plots (Figure 9) show a remarkable consistency with our DFT + U calculations. The theoretical model, employing Ud = 7 eV and Up = 8 eV, specifically predicts that V_Zn_ introduces localized band-tail/near-edge states near the band edges, facilitating visible-light transitions. This self-consistent agreement between quantitative composition analysis (EDX) and electronic structure modeling (DFT + U) provides a robust validation for assigning V_Zn_ as the primary origin of the visible-light photoresponse. While techniques such as XPS or PL could provide further localized chemical state information, the synergistic combination of spatially-resolved EDX and the DFT + U framework employed here offers a reliable identification of the defect nature. The energy levels of V_Zn_-induced states derived from our model perfectly align with the experimental PEC excitation wavelength (460 nm). Furthermore, V_Zn_ is a more plausible candidate under these non-equilibrium growth conditions compared to other intrinsic defects, which typically result in different electronic signatures.

## 4 Conclusion

In summary, this study demonstrates a visible-light-active PEC glucose sensor based on zinc-vacancy-rich ZnO nanorods fabricated on FTO via a simple electrodeposition route. While ZnO is classically regarded as a UV-only wide-band-gap oxide (Özgür et al., 2005), the results show that intrinsic V_Zn_ defects can activate a clear visible-light response without relying on complex doping procedures, noble-metal decoration, or multi-component heterostructures.

Experimentally, the electrodeposited ZnO NRs exhibit an optical band gap of ∼2.32 eV and deliver a stable glucose-dependent photocurrent under 460 nm illumination in phosphate buffer (pH 7.4), with a linear range of 0.5–10 mM and a detection limit of 12.5 μM. Mechanistically, spatially resolved EDX analyses along individual nanorods reveal a Zn-deficient compositional gradient consistent with enhanced VZn formation, while DFT + U density-of-states calculations confirm that V_Zn_ introduces band-tail/sub-bandgap states near the band edges that reduce the effective excitation energy and promote carrier generation and interfacial charge transfer during glucose oxidation.

Overall, the novelty of this work lies in identifying V_Zn_ as the primary driver for visible-light PEC sensitivity in ZnO nanorods and validating this defect-driven mechanism through a combined experimental–theoretical framework by correlating spatially resolved EDX compositional analysis and optical/PEC measurements with DFT + U electronic-structure calculations (band structure and DOS/PDOS), which reveal $V_{\text{Zn}}$ -induced band-tail/sub-bandgap states enabling visible-light photoexcitation. Beyond glucose sensing, these findings highlight a broader materials-design concept: intrinsic defect engineering can be exploited to extend the photoresponse of wide-band-gap semiconductors into the visible region in a scalable and metal-free manner, offering practical design principles for solar-compatible photoelectrochemical systems. Future studies will focus on the comprehensive analytical validation of mass transfer dynamics and matrix interference configurations to fully transition this defect-engineered platform into practical diagnostic applications.

## Data Availability

The raw data supporting the conclusions of this article will be made available by the authors, without undue reservation.

## References

[B1] Ahmad R. Tripathy N. Ahn M.-S. Bhat K. S. Mahmoudi T. Wang Y. (2017). Highly efficient non-enzymatic glucose sensor based on CuO modified vertically-grown ZnO nanorods on electrode. Sci. Rep. 7, 5715. 10.1038/s41598-017-06064-8 28720844 PMC5515932

[B2] Anisimov V. I. Zaanen J. Andersen O. K. (1991). Band theory and mott insulators: hubbard U instead of stoner I. Phys. Rev. B 44, 943–954. 10.1103/PhysRevB.44.943 9999600

[B3] Bakranova D. Seitov B. Bakranov N. (2023). Photocatalytic and glucose sensing properties of ZnO-Based nanocoating. ChemEngineering 7, 22. 10.3390/chemengineering7020022

[B4] Baruah S. Maibam B. Borah C. K. Agarkar T. Kumar A. Kumar S. (2021). A highly receptive ZnO-Based enzymatic electrochemical sensor for glucose sensing. IEEE Sens. J. 21, 14601–14608. 10.1109/JSEN.2021.3069303

[B5] Bo X. Bai J. Yang L. Guo L. (2011). The nanocomposite of PtPd nanoparticles/onion-like mesoporous carbon vesicle for nonenzymatic amperometric sensing of glucose. Sens. Actuators, B 157, 662–668. 10.1016/j.snb.2011.05.050

[B6] Chinnadayyala S. R. Park I. Cho S. (2018). Nonenzymatic determination of glucose at near neutral pH values based on the use of nafion and platinum black coated micronBedle electrode array. Microchim. Acta 185, 250. 10.1007/s00604-018-2770-1 29627889

[B7] Chitare Y. M. Jadhav S. B. Pawaskar P. N. Magdum V. V. Gunjakar J. L. Lokhande C. D. (2021). Metal oxide-based composites in nonenzymatic electrochemical glucose sensors. Industrial and Eng. Chem. Res. 60, 18195–18217. 10.1021/acs.iecr.1c03662

[B8] Dhara K. Mahapatra D. R. (2017). Electrochemical nonenzymatic sensing of glucose using advanced nanomaterials. Microchim. Acta 185, 49. 10.1007/s00604-017-2609-1 29594566

[B9] Dong Q. Ryu H. Lei Y. (2021). Metal oxide based non-enzymatic electrochemical sensors for glucose detection. Electrochimica Acta 370, 137744. 10.1016/j.electacta.2021.137744

[B10] Ghannam H. Bazin C. Chahboun A. Turmine M. (2018). CrystEngComm 20, 7420–7432. 10.1039/C8CE01223G

[B11] Guler A. C. Antos J. Masar M. Urbanek M. Machovsky M. Kuritka I. (2023). Comprehensive evaluation of photoelectrochemical performance dependence on geometric features of ZnO nanorod electrodes. Nanoscale Adv. 5, 3091–3103. 10.1039/D3NA00089C 37260485 PMC10228492

[B12] Haghparas Z. Kordrostami Z. Sorouri M. Rajabzadeh M. Khalifeh R. (2021). Highly sensitive non-enzymatic electrochemical glucose sensor based on dumbbell-shaped double-shelled hollow nanoporous CuO/ZnO microstructures. Sci. Rep. 11, 344. 10.1038/s41598-020-79460-2 33431992 PMC7801383

[B13] Haruna K. Salleh N. A. Deghfel B. Yaakob M. K. Mohamad A. A. (2020). Results in Phys. 16, 102829. 10.1016/j.rinp.2019.102829

[B14] He L. Zhang Q. Gong C. Liu H. Hu F. Zhong F. (2020). The dual-function of hematite-based photoelectrochemical sensor for solar-to-electricity conversion and self-powered glucose detection. Sens. Actuators, B 310, 127842. 10.1016/j.snb.2020.127842

[B15] Janotti A. Van de Walle C. G. (2007). Native point defects in ZnO. Phys. Rev. B 76, 165202. 10.1103/PhysRevB.76.165202

[B16] Janotti A. Van de Walle C. G. (2009). Fundamentals of zinc oxide as a semiconductor. Rep. Prog. Phys. 72, 126501. 10.1088/0034-4885/72/12/126501

[B17] Lany S. Zunger A. (2010). Many-bodyGWcalculation of the oxygen vacancy in ZnO. Phys. Rev. B 81, 113201. 10.1103/PhysRevB.81.113201

[B18] Larsen A. H. Mortensen J. J. Blomqvist J. Castelli I. E. Christensen R. Dułak M. (2017). The atomic simulation environment—a Python library for working with atoms. J. Phys.: Condens. Matter 29, 273002. 10.1088/1361-648X/aa680e 28323250

[B19] Lee C.-T. Chiu Y.-S. Ho S.-C. Lee Y.-J. (2011). Investigation of a photoelectrochemical passivated ZnO-based glucose biosensor. Sensors 11, 4648–4655. 10.3390/s110504648 22163867 PMC3231388

[B20] Li M. Wang H. Wang X. Lu Q. Li H. Zhang Y. (2019). Ti3C2/Cu2O heterostructure based signal-off photoelectrochemical sensor for high sensitivity detection of glucose. Biosens. Bioelectron. 142, 111535. 10.1016/j.bios.2019.111535 31376715

[B21] Mao X. Zhang C. (2022). A microfluidic cloth-based photoelectrochemical analytical device for the detection of glucose in saliva. Talanta 238, 123052. 10.1016/j.talanta.2021.123052 34808571

[B22] Naikoo G. A. Salim H. Hassan I. U. Awan T. Arshad F. Pedram M. Z. (2021). Recent advances in non-enzymatic glucose sensors based on metal and metal oxide nanostructures for diabetes Management- A review. Front. in Chem. 9, 748957. 10.3389/fchem.2021.748957 34631670 PMC8493127

[B23] Nguyen T. N. H. Jin X. Nolan J. K. Xu J. Le K. V. H. Lam S. (2020). Printable nonenzymatic glucose biosensors using carbon Nanotube-PtNP nanocomposites modified with AuRu for improved selectivity. ACS Biomater. Sci. Eng. 6, 5315–5325. 10.1021/acsbiomaterials.0c00647 33455280 PMC8203305

[B24] Oba F. Togo A. Tanaka I. Paier J. Kresse G. (2008). Defect energetics in ZnO: a hybrid hartree-fock density functional study. Phys. Rev. B 77, 245202. 10.1103/PhysRevB.77.245202

[B25] Ogurtsova K. Guariguata L. Barengo N. C. Ruiz P. L.-D. Sacre J. W. Karuranga S. (2022). IDF diabetes atlas: global estimates of undiagnosed diabetes in adults for 2021. Diabetes Res. Clin. Pract. 183, 109118. 10.1016/j.diabres.2021.109118 34883189

[B26] Özgür Ü. Alivov Y. I. Liu C. Teke A. Reshchikov M. A. Doğan S. (2005). Prog. Mater. Sci. 50, 465–563. 10.1016/j.pmatsci.2008.07.002

[B27] Persson C. Zunger A. (2007). Appl. Phys. Lett. 91, 266101. 10.1063/1.2794380

[B28] Quantum ESPRESSO. (2026). Quantum ESPRESSO: pw.x input description (INPUT_PW). Available online at: https://www.quantum-espresso.org/Doc/INPUT_PW.html (Accessed April 12, 2026)

[B29] Siavash Moakhar R. Mirzaei M. Elizabeth Flynn S. Jalali M. Sanati A. Mahshid S. (2024). Photoelectrochemical sensing of titanium oxide nanostructures for the detection of glucose: fabrication methods and signal enhancement strategies. Microchem. J. 201, 110528. 10.1016/j.microc.2024.110528

[B30] Singer N. Pillai R. G. Johnson A. I. D. Harris K. D. Jemere A. B. (2020). Nanostructured nickel oxide electrodes for non-enzymatic electrochemical glucose sensing. Microchim. Acta 187, 196. 10.1007/s00604-020-4171-5 32125544

[B31] Tao B. Li X. Miao F. Zhang P. Gao B. Yang W. (2023). Non-enzymatic photoelectrochemical sensor based on rGO/NiCo_2_O_4_/ZnO for glucose detection. IEEE Trans. Electron Devices 70, 4366–4371. 10.1109/TED.2023.3289786

[B32] Tobaldi D. M. Espro C. Leonardi S. G. Lajaunie L. Seabra M. P. Calvino J. J. (2020). Photo-electrochemical properties of CuO–TiO_2_ heterojunctions for glucose sensing. J. Mater. Chem. C 8, 9529–9539. 10.1039/D0TC01975E

[B33] Wang C. Yang Z. Lv J. Zhu Q. Jiang J. Zhao M. (2019). Surface morphology, electrochemical and electrical performances of ZnO thin films sensitized with Ag nanoparticles by UV irradiation. J. Mater. Sci.: Mater. Electron. 30, 9798–9805. 10.1007/s10854-019-01316-x

[B34] Yang Y. Yan K. Zhang J. (2017). Dual non-enzymatic glucose sensing on Ni(OH)2/TiO2 photoanode under visible light illumination. Electrochim. Acta 228, 28–35. 10.1016/j.electacta.2017.01.050

[B35] Ye Z. Miao F. Tao B. Zang Y. Chu P. K. (2021). Facile synthesis of ZnO doped with Au nanoparticles for sensitive and reliable photoelectrochemical detection of glucose. Ionics 27, 4449–4459. 10.1007/s11581-021-04198-4

[B36] Yoo D.-H. Cuong T. V. Lee S. Hwang W. S. Yoo W. J. Hong C.-H. (2014). J. Phys. Chem. C 118, 21969–21977. 10.1021/jp5039046

[B37] Zheng Z. Lim Z. S. Peng Y. You L. Chen L. Wang J. (2013). General route to ZnO nanorod arrays on conducting substrates via galvanic-cell-based approach. Sci. Rep. 3, 2434. 10.1038/srep02434 23942316 PMC3743057

[B38] Zhou F. Li Y. Tang Y. Gao F. Jing W. Du Y. (2022). A novel flexible non-enzymatic electrochemical glucose sensor of excellent performance with ZnO nanorods modified on stainless steel wire sieve and stimulated via UV irradiation. Ceram. Int. 48, 14395–14405. 10.1016/j.ceramint.2022.01.332

