# Correction: Zinc-vacancy defects in ZnO nanorods induced visible-light activity of photoelectrochemical glucose sensing: experimental and DFT+U analysis

**DOI:** 10.3389/fchem.2026.1997693

**Published:** 2026-09-24

**Authors:** Trung Tin Tran, Huynh Nhu Nguyen Thi, Trung Nghia Tran, Tran Viet Cuong, Quang Vinh Lam

**Affiliations:** 1 Faculty of Physics and Physics Engineering, University of Science, VNUHCM, Ho Chi Minh City, Vietnam; 2 Laboratory of Laser Technology, Faculty of Applied Science, Ho Chi Minh City University of Technology (HCMUT), Ho Chi Minh City, Vietnam; 3 Vietnam National University Ho Chi Minh City, Ho Chi Minh City, Vietnam; 4 VKTECH Research Center, NTT Hi-Tech Institute, Nguyen Tat Thanh University, Ho Chi Minh City, Vietnam; 5 Nguyen Tat Thanh University Center for Hi-Tech Development, Saigon Hi-Tech Park, Ho Chi Minh City, Vietnam

**Keywords:** DFT+U, electrodeposition, electronic structure, non-enzymatic glucose sensor, photoelectrochemical, ZnO nanorods

The Acknowledgments statement was erroneously omitted. The statement should read:

“We acknowledge the support of time and facilities from Ho Chi Minh City University of Technology (HCMUT), VNU-HCM for this study.”

The original version of this article has been updated.

